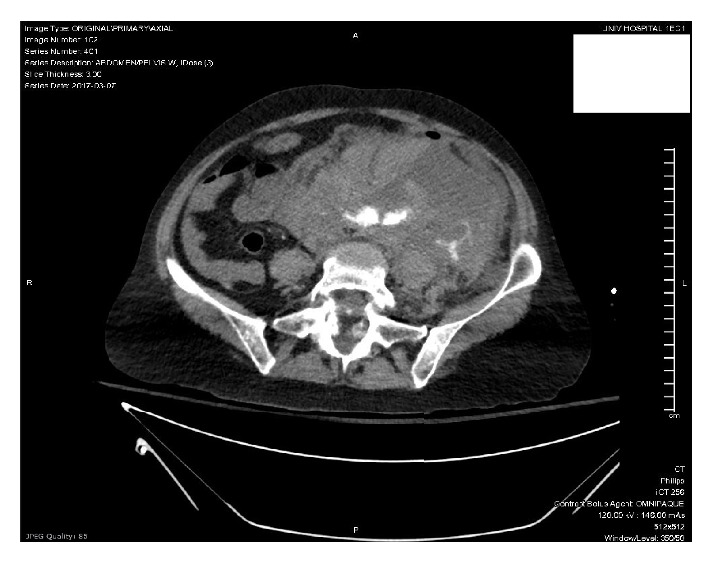# Two Cases of Catastrophic AAA Rupture in Young Women with Systemic Lupus Erythematosus

**DOI:** 10.1155/2018/1049568

**Published:** 2018-09-16

**Authors:** David Noorvash, Kevin King, Meera Gebrael

**Affiliations:** The Department of Emergency Medicine, University of Texas Health San Antonio, San Antonio, TX, USA

## Abstract

We present two cases of young women with a past medical history significant for systemic lupus erythematosus (SLE), who presented to the Emergency Department with a ruptured abdominal aortic aneurysm (AAA). These cases are of particular interest because the patients did not fit the typical demographic for patients who present with a ruptured AAA. Based on these cases and a review of the relevant literature, ED providers should maintain a higher index of suspicion for AAA rupture in patients with autoimmune diseases, especially SLE.

## 1. Introduction

A ruptured abdominal aortic aneurysm (AAA) is a catastrophic event that carries a very high mortality. Prompt diagnosis and management are imperative to improve outcomes in this very time-sensitive condition. [1] The cases presented here are of particular interest because the patients involved do not fit the classic demographic for a ruptured AAA. Both were young women in their 40s. Although there are several case reports in the vascular and cardiothoracic surgery literature that suggest an association between SLE and AAA development, [2] there is a lack of similar reports in the Emergency Medicine literature. Young patients are at greater risk for a delayed or missed diagnosis leading to poor outcomes. Emergency physicians should maintain a higher index of suspicion for aortic aneurysms in any individual with SLE, irrespective of age, who presents with symptoms such as severe abdominal, flank, back pain, syncope, or gastrointestinal bleeding.

## 2. Case Presentations

### 2.1. Case #1

A previously well 41-year-old woman, with a past medical history significant only for SLE, presented to the Emergency Department with acute onset of severe LLQ abdominal pain radiating to her left flank and back. Her pain began abruptly approximately 5 hours prior to her arrival and started while she was resting in bed. She denied any nausea or vomiting but did endorse several days of diarrhea with dark-appearing stools. She had been PO tolerant earlier in the evening and was feeling fine until her pain started. The patient reported that she had not sought regular medical care for at least several months prior to this visit and did not take any medications, smoke, or use illicit substances.

On her initial presentation and physical exam, the patient was in extremis, writhing in pain on the bed. She was noted to have diffuse abdominal tenderness to palpation and severe left flank pain, with guarding and rigidity. Her initial vital signs were stable, with a heart rate of 70 and blood pressure 127/84.

While awaiting her CT scan, the patient suddenly became unresponsive and lost pulses. CPR was briefly initiated and the patient quickly regained pulses and awoke. At this time, she was noted to be pale, diaphoretic, tachycardic, and profoundly hypotensive. She was rapidly intubated and resuscitated with several liters of crystalloid, and vasopressors were initiated due to refractory hypotension. A Cordis central catheter was placed, and a massive transfusion protocol was initiated.

**Figure 1:**
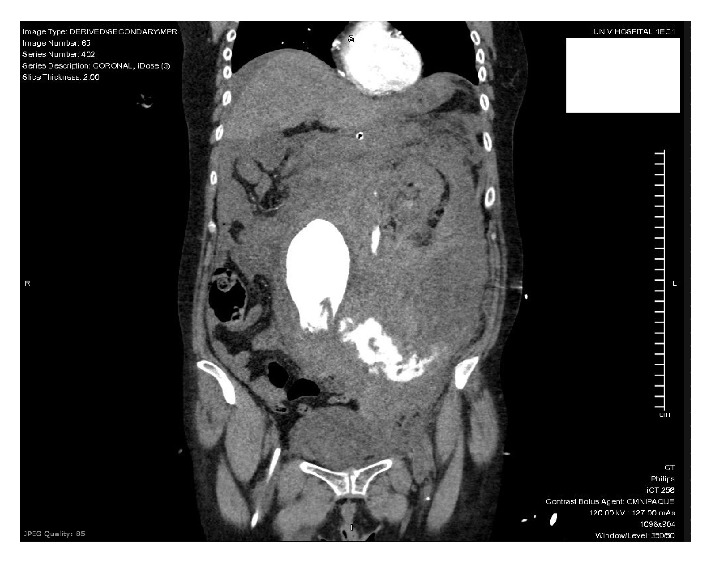


**Figure 2:**
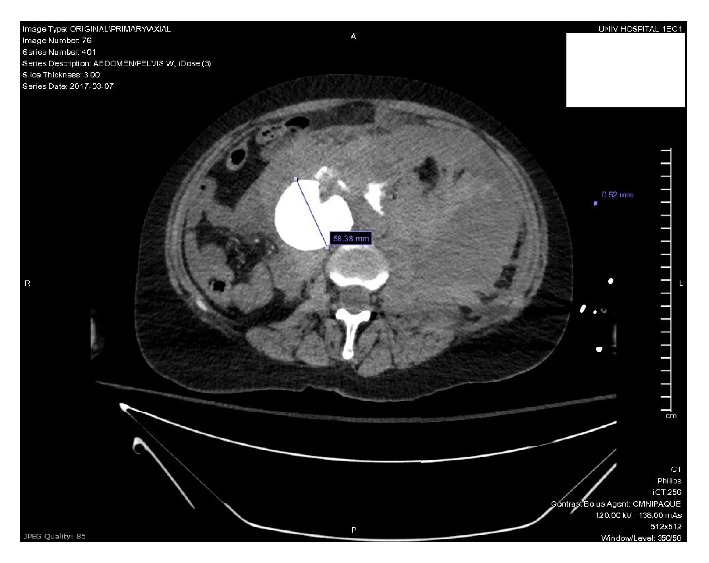


**Figure 3:**
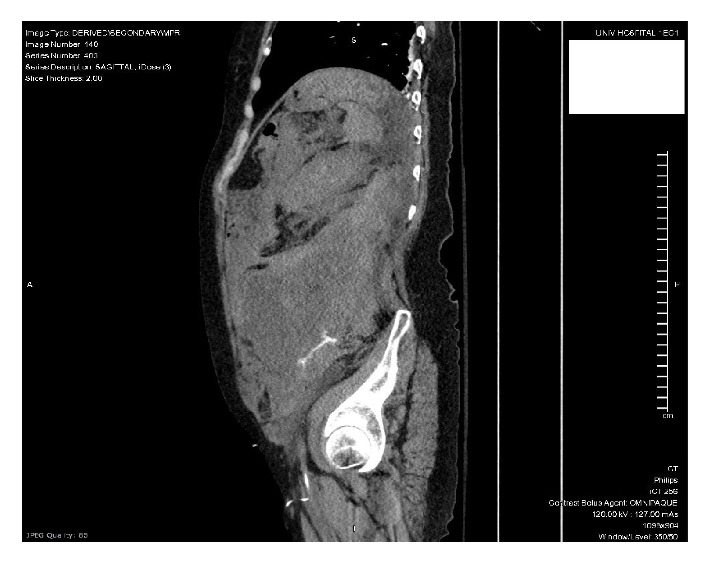


Once the patient was stable enough to obtain a CT scan, she was found to have a large, ruptured 5.8cm abdominal aortic aneurysm with active extravasation (Figures 1 and 2), extensive hemoperitoneum, and a large left-sided retroperitoneal hematoma (Figures 3 and 4). She also had nonvisualization of the bilateral common iliac arteries, the celiac artery, and the superior mesenteric artery (SMA).

Significant laboratory findings included an initial hemoglobin/hematocrit of 11.2/34.1 that acutely dropped to 7.8/23.4 over the course of 1 hour; D-dimer > 7360; INR 1.4; lactate 11.7; and a pH of 6.88.

The patient was taken to the operating room by the vascular surgery team for definitive management. However, despite cross-clamping of the aorta and continued massive transfusion of blood products, the patient ultimately lost pulses and expired after several additional rounds of CPR.

### 2.2. Case #2

This patient was a 47-year-old woman—with a past medical history of SLE, a known stable AAA, previous left SFA-to-peroneal bypass, HTN, HLD, DM, and CKD—who presented to the Emergency Department with acute-onset of periumbilical abdominal pain. She endorsed nausea and nonbloody emesis and denied any radiation of her pain.

The patient was scheduled for routine follow-up the following day with her vascular surgeon for her known AAA, which was measured at 4.5cm six months prior. Her AAA had yet to be operated on and had been managed conservatively with medical management of her comorbidities. She took warfarin “for lupus,” and it is unclear whether this was due to an associated hypercoagulable state such as antiphospholipid antibody syndrome. She was a nonsmoker and did not use any illicit substances.

Shortly following her presentation to the ED, the patient suddenly lost consciousness. She was immediately taken to the medical resuscitation bay, where she was unresponsive with depressed respirations. Mechanical ventilation was initiated and an eFAST ultrasound revealed a small amount of free fluid in the pelvis.

The patient subsequently lost pulses, and CPR was immediately initiated. Two liters of normal saline were administered, epinephrine was given, and crash blood was administered. A repeat FAST exam showed accumulating blood within the peritoneum and the patient's abdomen was noted to be growing progressively distended. Significant laboratory abnormalities included an H/H of 4.9/15.8, INR 1.7, and Creatinine of 1.9.

A massive transfusion protocol was initiated and multiple specialty services were called to the bedside, including trauma surgery, vascular surgery, and cardiology. However, despite maximal resuscitative efforts, the patient failed to regain pulses and was declared deceased after 58 minutes of aggressive resuscitation.

## 3. Discussion

The pathogenesis of abdominal aortic aneurysm formation typically involves a progressive thinning of the media layer of the vascular wall, via progressive decreases in elastin, collagen, and fibrolamellar units [1]. The progressive thinning decreases the tensile strength of the aortic wall, causing it to dilate, which weakens the wall and increases the force on the wall, leading to further dilatation [1]. An aortic aneurysm is defined as any dilatation greater than 3 cm, and repair is generally recommended for diameters greater than 5 cm.

Ruptured aortic aneurysms have a variable presentation and are commonly mistaken for other disease processes on initial presentation. Retroperitoneal ruptures can tamponade due to the limited potential space of the retroperitonium, reducing the amount of hemorrhage and leading to a transient stabilization in a patient's vital signs. In the operating room, opening of the peritoneal cavity sometimes releases this pressure, leading to massive exsanguination and death. The mortality without surgery approaches 100%, and there is still a 50% mortality for patients who do make it to the operating room.

Classically, aortic aneurysm is thought of as a condition for the old. Yet, several studies suggest an association between autoimmune disease, specifically SLE, and aortic aneurysm formation [2]. Among individuals with aortic aneurysms, the average age of diagnosis is 30 years younger in patients with SLE when compared to the general population [3]. In two large retrospective studies, patients with SLE had a two-to-five-fold increased risk of aortic aneurysms when compared to controls matched for age and gender [4, 5]. Two additional large studies also revealed an increased prevalence of AAA in conjunction with other autoimmune diseases, such as rheumatoid arthritis [6, 7].

Multiple pathways likely explain this observed association between SLE and aortic aneurysm formation. SLE is associated with the development of atherosclerosis and vasculitis, both of which confer an increased risk of aneurysmal development [8]. Inflammation and vasculitis associated with SLE can result in weakening of the vascular wall, leading to aneurysm formation [2]. Vasculitis in SLE patients is more likely to lead to thoracic aneurysms, while atherosclerosis more commonly causes abdominal aneurysms, which are more likely to be fatal. Moreover, SLE-induced damage of renal vasculature can lead to hypertension [4], an independent risk factor for aneurysmal development. Prolonged steroid use, which is often present in SLE patients, is also independently associated with the development of atherosclerosis and vascular disease and is likely to confer additional risk in these patients [3, 4].

The two cases presented here involve relatively young women who presented to the ED with a ruptured AAA. Typically, patients who present with AAA have classic risk factors such as male gender, advanced age, smoking, a positive family history, and connective tissue disorders such as Marfan's disease [1]. Autoimmune conditions such as SLE are perhaps less commonly thought of as risk factors. We were unable to locate any other case reports in the Emergency Medicine literature correlating autoimmune disease such as SLE with a ruptured AAA, despite multiple reports in the vascular surgery literature. [2].

These cases illustrate why ED physicians should have a higher index of suspicion for AAA when treating patients with SLE, in order to avoid delaying or missing this crucial, time-sensitive diagnosis. Furthermore, perhaps there should be increased screening for AAA in SLE patients via inexpensive and noninvasive methods such as point-of-care ultrasound. In SLE patients with known AAA, more frequent surveillance and earlier surgical intervention may also be warranted.

## Figures and Tables

**Figure 4:**